# The Hungry Daemon: Does an Energy-Harvesting Active Particle Have to Obey the Second Law of Thermodynamics?

**DOI:** 10.3390/e27090918

**Published:** 2025-08-30

**Authors:** Simon Bienewald, Diego M. Fieguth, James R. Anglin

**Affiliations:** 1 Fachbereich Mathematik, Humboldt-Universität zu Berlin, D-10099 Berlin, Germany; 2 State Research Center OPTIMAS and Fachbereich Physik, RPTU Kaiserslautern-Landau, D-67663 Kaiserslautern, Germany

**Keywords:** Second Law of Thermodynamics, statistical thermodynamics, nonlinear coupling, active particle physics, energy harvesting

## Abstract

Thought experiments like Maxwell’s Demon or the Smoluchowski–Feynman Ratchet can help in pursuing the microscopic origin of the Second Law of Thermodynamics. Here we present a more sophisticated mechanical system than a ratchet, consisting of a Hamiltonian (non-Brownian) active particle which can harvest energy from an environment which may be in thermal equilibrium at a single temperature. We show that while a phenomenological description would seem to allow the system to operate as a Perpetual Motion Machine of the Second Kind, a full mechanical analysis confirms that this is impossible, and that perpetual energy harvesting within a mechanical system can only occur if the environment has an energetic population inversion similar to a lasing medium.

## 1. Introduction: Microphysics and the Second Law

The Laws of Thermodynamics are in no doubt, but the relationship of the Second Law to microscopic physics is still not fully clear. The Second Law cannot really be an independent axiom of causality in its own right, since deterministic mechanics already prescribes everything that can ever happen, and never requires any electron to consult thermodynamics in order to learn what to do next. General proof of the Second Law as a theorem within mechanics remains elusive, however. In particular the thermodynamical “arrow of time”, with entropy increasing only in the future direction, is still not easy to reconcile with microscopic time-reversal symmetry (see the Loschmidt paradox [1,2,3]), or with Poincaré recurrence [4]. It still seems possible that thermodynamics is *not* only due to microscopic equations of motion, but may instead reflect a constraint on the initial conditions of the universe.

The root of our difficulties in understanding the connection between Thermodynamics and microphysics lies in the fact that systems in which Thermodynamics is of practical importance are generally large, in the sense of having many degrees of freedom. Even systems as small as micro-organisms or molecular machines have so many moving parts that fully microscopic description is unfeasible. We can still describe such systems with simple models, but we can generally only do so by using effective equations of motion with dissipation and noise terms, which explicitly violate the unitarity of microscopic quantum evolution or, in the classical limit, Liouville’s theorem. It has to be emphasized that such non-unitary terms are not fundamental, but rather artifacts of truncating a full, microscopic description of a system into a partial, macroscopic description. Macroscopic descriptions can certainly be both accurate and useful, but for understanding the fundamental relationship between Thermodynamics and microphysics they are problematic, since they inherently take for granted some of the very things we are trying to investigate, such as relaxation and thermalization. The validity of the macroscopic description may not be in doubt, but to understand why it is valid, we cannot start by assuming it.

Important insights like fluctuation theorems [5,6,7] have been obtained within the macroscopic framework of non-equilibrium statistical mechanics by keeping the models of dissipation and noise as simple as possible while analyzing key parts of the system microscopically. We do not attempt a proper survey of even a narrow section of this literature, however, because in this paper, in contrast, we take the extreme approach of not including dissipation or noise at all: we study a fully microscopic classical system which obeys Liouville’s theorem, and has only a few degrees of freedom. By exploiting a certain feature of nonlinear dynamics (an isolated Chirikov resonance [8]), our purely mathematical Hamiltonian system has nonetheless previously been shown to behave like a combustion engine [9] or active particle [10], while exhibiting non-trivial constraints, due to adiabatic effects that resemble Thermodynamics.

Our approach cannot be a false one in principle, because there is no doubt that any real active particle could run for an arbitrarily long time within a sufficiently large isolated box containing some large but finite number of particles, whether these were the water molecules and nutrients supporting a micro-organism or the volumes of coal and air for running a steam locomotive. The entire finite, isolated dynamical system inside that box would obey unitarity, and Liouville’s theorem in the classical limit, but it would surely also obey Thermodynamics in its most general form (not necessarily equilibrium). Demonstrating this, and fully understanding how it works, is the outstanding problem of relating Thermodynamics to microphysics. We tackle this problem head-on, albeit in a very simple limit in which the finite, isolated system is actually not large but small.

In particular we will present a model mechanical system (that is, a mathematical Hamiltonian model) which is on the one hand small and simple enough to analyze microscopically in its entirety, and yet which on the other hand directly confronts the Kelvin–Planck formulation of the Second Law forbidding Perpetual Motion Machines of the Second Kind. In this sense our Liouvillian model can be considered as a thought experiment in the tradition of Maxwell’s Demon [11,12] and the equilibrium Smoluchowski–Feynman Ratchet [13,14], which propose to violate the Second Law of Thermodynamics by using simple mechanics.

## 2. Demons, Ratchets, and Active Particles

Our model will consist, firstly, of a *non-Brownian active particle* [10]: a particle which can move against an external force by drawing on an internal energy supply (a ‘depot’) [15,16,17,18,19]. Active particles in this sense have been studied extensively as idealized models for mobile micro-organisms or nano-motors; here we adapt the active particle concept to a different purpose. On the one hand, we eliminate the viscous drag and Brownian noise which are usually considered for active particles, since they are necessary for modelling systems like bacteria swimming through water, but would be undesirable macroscopic intrusions in our quest for microscopic understanding. And on the other hand, we define a specific microscopic mechanism, so that the transfer of energy from our particle’s depot into its active motion occurs under standard Hamiltonian equations of motion, rather than in a phenomenological energy budget equation, which simply assumes that the depot supplies energy for motion somehow, through some complicated biological machinery.

As shown in [9], our active particle certainly cannot move against the external force forever all by itself, because its energy depot is finite. In this paper, however, we will allow the active particle to replenish its internal depot by interacting with localized degrees of freedom as it moves past them. Energy harvesting by active particles has also been studied in dissipative models intended to represent biological systems [20,21,22,23], but here again we will define a non-dissipative form of energy harvesting, since our goal is not biological modelling but fundamental microphysics. Our food-like localized degrees of freedom (hereafter ‘crumbs’) will be considered as part of our whole system, along with the active particle—no single one of our degrees of freedom will obey Liouville’s theorem separately from the others, but the entire system of all of them will obey it in the larger total phase space. The localized crumb degrees of freedom will not be noisy in time, but they will have randomly distributed initial energies—in exactly the same way that individual gas molecules have randomly distributed energies when, one after another, they meet Maxwell’s Demon [11,12], or collide with a propeller blade in a Smoluchowski–Feynman Ratchet [13,14].

The energies of the individual molecules which Maxwell’s Demon or the Ratchet successively encounter are in particular distributed in a canonical ensemble, and so the set of all of the molecules makes up a spatially distributed thermal reservoir that is in equilibrium with a single well-defined temperature. In exactly the same way, the initial energies of our localized ‘crumb’ degrees of freedom will be distributed in a canonical ensemble so that they also constitute an equilibrium reservoir with a given temperature. In particular our individual crumbs will be rotors, and an ensemble of rotors or spins with thermally distributed energy is a common concrete model for a heat bath. If necessary, the validity of the set of crumbs as a thermal reservoir can be secured by postulating further interactions among the crumbs—or between the crumbs and an additional reservoir—that are weak enough to neglect over all time scales we consider, yet sufficient to make the whole set equilibrate ergodically over longer times.

So, although on the one hand we have a dynamical problem within Liouvillian mechanics, as our simple Hamiltonian active particle interacts one at a time with a succession of simple Hamiltonian crumbs, nevertheless on the other hand we also have a problem to which Thermodynamics applies, just as it does to the Demon and the Ratchet, as our active particle seeks to keep doing work (moving against a force) by drawing energy from a depot which is itself drawing from a thermal reservoir (the set of all crumbs). To the question of whether the active particle can keep moving against the external force indefinitely by harvesting energy from a thermal environment, mechanics and Thermodynamics both offer answers. And, as with the Demon and the Ratchet, so in our case does physical intuition offer an answer as well.

Intuition conflicts with Thermodynamics in our model just as it does for the Ratchet and Demon: it is initially hard to see why our energy-harvesting active particle will not be able to keep moving perpetually against the external force, as long as the temperature of the crumbs is sufficiently high that they can supply enough energy. The crumbs are a reservoir at a given temperature, and the energy depot of the active particle interacts with them; one expects that the depot will tend to stay thermalized with the crumbs so that its energy cannot fall too far below their average energy, and it will thus keep powering the active motion perpetually. Thermodynamics nonetheless says that the particle cannot do work perpetually like that, no matter how much energy the crumbs seem to offer, because this would violate the Kelvin–Planck formulation of the Second Law [24]: “It is impossible to devise a cyclically operating device, the sole effect of which is to absorb energy in the form of heat from a single thermal reservoir and to deliver an equivalent amount of work.” Although it can be subtle to define work in general, steady motion against an external force must count as work, if anything does. The power that drives the active particle’s motion is drawn directly from its depot, and if its depot can be replenished indefinitely from a single thermal environment, then a perpetually moving active particle must be a cyclically operating device of precisely the kind that is forbidden by the Second Law—a Perpetual Motion Machine of the Second Kind.

Historical efforts to vindicate Thermodynamics against the challenges of Maxwell’s Demon, recently surveyed in [12], have needed to unravel subtle questions of information processing (or even intelligent agency) on the part of the Demon, and of the entropy that may be carried by the medium which the Demon uses to detect the molecules. Understanding the Smoluchowski–Feynman Ratchet also requires careful study of the role of friction [17] in letting a pawl stop a sprocket from turning backwards, and of the Brownian motion of the pawl itself as an independent degree of freedom. In our case, in contrast, we will not need to consider information, dissipation, or noise to show that mechanics unambiguously supports Thermodynamics, and not intuition. The price for this will be that our mechanical system is somewhat more complicated than a sliding door or a ratchet.

## 3. Hamiltonian Daemon

Our mathematical Hamiltonian model for energy harvesting by an active particle is based on a previous mechanical model for a non-Brownian active particle. The *Hamiltonian Daemon* is a mechanical model introduced in [9] which is able to transfer energy from fast to slow degrees of freedom using a nonlinear resonance. (The model is referred to as a “daemon” rather than a “demon” in order to avoid an implicit claim that the model is a Demon like Maxwell’s; instead the name is in analogy with the small autonomous processes in Unix-family computer operating systems.) In [25] it was shown how this system has the properties of an active particle: It can sustain motion against external forces by drawing energy from an internal depot.

In [10] it was further shown that this mechanical model can still operate as an active particle under strong damping with Eulerian motion, and so it is not necessarily incompatible with the more usual kind of Brownian active particle; in this paper we include no dissipation or noise because they would be obstacles to our purpose, as discussed above. To focus on the problem of energy harvesting within Liouvillian mechanics, we will consider here the undamped Daemon, and add to it interactions with crumbs that will allow this “hungry daemon” to “feed”. We first review the “Daemon” model itself.

### 3.1. The Daemon Hamiltonian

The Hamiltonian of the Daemon model of [9] is a particular case of the general form

(1) $$\begin{array}{c} H_{D}=H_{M}+H_{I}+\gamma H_{C}, \end{array}$$

where $H_{M}$ is the motional energy (kinetic and potential) of the particle, $H_{I}$ is the particle’s internal energy depot, and $H_{C}$ describes the coupling between the previous two subsystems. The parameter γ is in general small so that a potentially large amount of “fuel” energy can be converted gradually, over a long time, into a large amount of work in moving the particle against an opposing force. Making γ large would change the Daemon from an engine-like system into something more like a bomb.

The particle’s motional energy $H_{M}$ includes the usual kinetic energy with a mass *M*, plus a potential with dependence on position *q* that can always be taken as linear over some range. The depot is modelled as an integrable subsystem with some high frequency Ω, described in canonical action-angle variables (δ,I). The Daemon’s particular case of (1) thus includes

(2) $$\begin{array}{cc} H_{M}\left(q,p\right) & =\frac{p^{2}}{2M}+Mgq, \end{array}$$

(3) $$\begin{array}{cc} H_{I}\left(\delta ,I\right) & =\Omega I, \end{array}$$

where *q* is the particle’s position, *p* its momentum and the action variable *I* of the depot is the “fuel level”, while the angular variable δ is the phase that is canonically conjugate to the action variable *I*.

We take the potential as exactly linear in *q* for simplicity; all of our discussion can be adapted to slowly varying potentials adiabatically, and cases where the opposing force is frictional rather than conservative are not qualitatively different [10,25]. For simplicity we also take the dependence of $H_{I}$ on *I* to be exactly linear, since our analysis likewise generalises adiabatically to (sufficiently weak) nonlinear dependence on *I*.

The energy depot must have a finite capacity, and thus the fuel level is bounded by a minimum and maximum fuel level $\pm I_{0}$. These upper and lower bounds on *I* must be respected by the coupling term $H_{C}$, which will make *I* time-dependent but can never make $|I|>I_{0}$.

### 3.2. The Nonlinear Coupling

**Figure 1:**
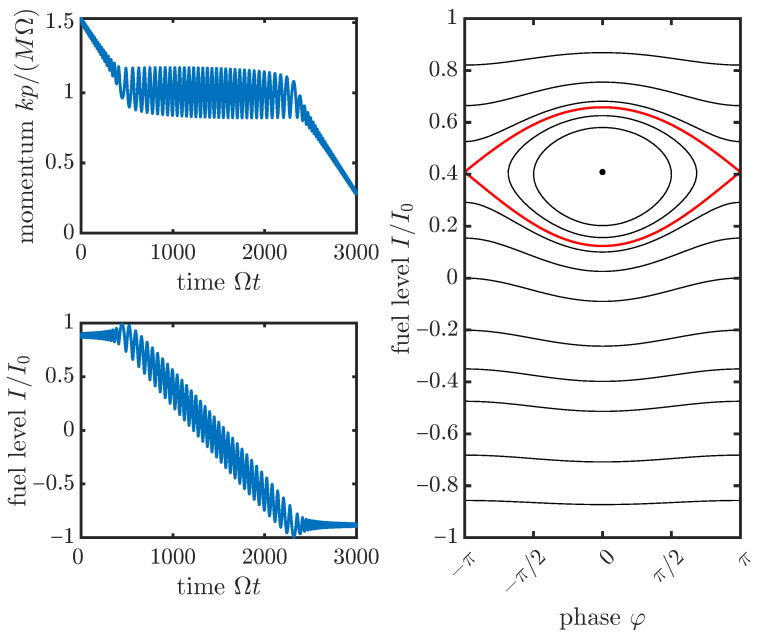
(**Left**): Typical trajectory of the active particle’s momentum *p* and fuel level *I* according to (1) over time during the active phase. The active phase is characterized by a steady energy flow from the particle’s fuel tank degree of freedom *I* to its momentum *p* into work against an external opposing force. During this process, the fuel level decreases at a nearly constant rate, while the momentum is held at a nearly constant value. (**Right**): Phase space of the transformed coordinate (φ,I) with φkq−δ with instantaneous contours at Ωt=1000. The red contour shows the separatrix of the system in that instant, which is the boundary in phase space between the active and the inactive phases of the particle’s dynamics. We chose here $Mg/(k\Omega I_{0})=0.001$, γ/Ω=0.02, $M\Omega /(k^{2}I_{0})=1$, $I\left(0\right)/I_{0}=0.9$, kp(0)/(MΩ)=1.5.

This requirement motivates the particular form of nonlinear coupling between the particle’s position *q* and its depot’s angle variable δ from [9], which can be obtained by transforming a system of interacting particles:

(4) $$\begin{array}{c} H_{C}\left(q,p,\delta ,I\right)=\sqrt{I_{0}^{2}-I^{2}}\cos\left(kq-\delta \right), \end{array}$$

for some constant *k*. This coupling provides a *Chirikov resonance* [8] which supports steady energy transfer between $H_{I}$ and $H_{M}$, but only in the narrow region of phase space where the argument of the cosine is approximately constant in time, i.e., $k\dot{q}-\dot{\delta }=0$. This condition defines the critical velocity $v_{c}=\Omega /k$ at which the active particle can steadily move, against the opposing force −Mg, by steadily drawing energy from its depot. Figure 1 shows a typical trajectory of the active particle’s momentum *p* and fuel level *I* during the active phase (as well as slightly before and after it).

Further details of the Daemon system’s behavior are supplied in [9,10,25]. For our current problem, the most essential feature of the Daemon’s active motion is that it eventually stops.

### 3.3. End of Active Motion

Since the internal depot energy is finite, active motion powered by the depot cannot continue forever. The secular rate at which *I* falls, during active motion, is exactly the rate $\dot{I}=-Mg/k$ at which the loss of internal depot energy ${\dot{H}}_{I}=-Mg\Omega /k$ provides the power $\left(p_{c}/M\right)\left(Mg\right)=+Mg\Omega /k$ to keep moving at speed $p_{c}/M$ against the external force −Mg. The longest time for which the particle can continue to move actively is thus $t_{m}=2kI_{0}/\left(Mg\right)$.

In [9] it is shown that a certain adiabatic invariant generally requires the active phase to end before *I* falls all the way to $-I_{0}$; it ends instead at some threshold $I\to I_{*}$ satisfying $-I_{0}<I_{*}<0$. This negative threshold $I_{*}$ is determined by the initial value of *I* (in fact $I_{*}≐-I_{i}$ in the absence of viscous drag on the particle’s motion). In [10] it is found that adding viscous drag against the active particle’s motion actually lets the active phase persist longer, possibly until the depot is fully drained, by lowering $I_{*}$. With or without drag, however, it is easy to confirm by integrating the equations of motion numerically that the only thing which brings the active phase to an end is *I* falling to some finite negative threshold.

This suggests that if *I* can simply be held above this negative threshold, by somehow restocking the energy depot as the particle moves, then the active phase could continue forever. It also seems intuitive that if the active particle interacts with crumbs that have more energy than it does, then it must on average be the winner in energy exchange with the crumbs, and thus be able to keep its depot energy above the threshold for continued active motion. Since this intuitive picture allows a Perpetual Motion Machine of the Second Kind, however, something must go wrong with it microscopically. To see what can go wrong, we introduce a mechanical model for crumbs that can exchange energy with the Daemon’s depot.

## 4. Microscopic Harvesting Model: Feeding from Crumbs

### 4.1. The Hungry Daemon Hamiltonian

We consider this total Hamiltonian with “crumbs” with which the “Hungry Daemon” active particle can interact:

(5) $$\begin{array}{cc} H & =H_{D}+\sum_{n}H_{n} \end{array}$$

(6) $$\begin{array}{cc} H_{n} & =\Omega J_{n}\\ + & \kappa \;\theta (a/2-|q_{n}-q\left|\right)\sqrt{\left(J_{0}^{2}-J_{n}^{2}\right)\left(I_{0}^{2}-I^{2}\right)}\cos\left(\delta -\beta_{n}\right), \end{array}$$

where $H_{D}$ is still the Daemon Hamiltonian of (1), and each new canonical pair of action-angle variables $\left(\beta_{n},J_{n}\right)$ represents a crumb at fixed location $q_{n}$, with $|J_{n}|\leq J_{0}$ for some $J_{0}$, in the same way that $\left|I\right|\leq I_{0}$. Each crumb *n* is a small version of the active particle’s internal depot; we will let all the crumbs have the same frequency Ω as the Daemon’s internal depot, because this resonant case seems likely to be optimal for energy harvesting. The step function $\theta (a/2-|q-q_{n}|)$ means that the active particle only interacts with each crumb if it is within a distance a/2 from that crumb—and for simplicity we will ensure that no crumbs are within a distance *a* from each other. The strength of the coupling between the active particle and the crumbs is given by κ. The resulting equations of motion are given in Appendix A.

The particular form of the coupling term in $H_{n}$ is chosen because it is equivalent to a classical spin–spin coupling $\propto (L_{x}L_{nx}+L_{y}L_{ny})$ in the minimal canonical representation of angular momentum

(7) $$\begin{array}{cc} L & =\left(\begin{array}{c} \sqrt{I_{0}^{2}-I^{2}}\cos\left(\delta \right)\\ \sqrt{I_{0}^{2}-I^{2}}\sin\left(\delta \right)\\ I \end{array}\right),\;\;L_{n}=\left(\begin{array}{c} \sqrt{J_{0}^{2}-J_{n}^{2}}\cos\left(\beta_{n}\right)\\ \sqrt{J_{0}^{2}-J_{n}^{2}}\sin\left(\beta_{n}\right)\\ J_{n} \end{array}\right). \end{array}$$

Although our numerical results in this paper will all now be based entirely on this specific form of coupling, as well as on the simplifying assumptions of equal parameters $J_{0}$, Ω, κ, and *a* for all crumbs, we will argue in our next Section that our conclusions are generic.

**Figure 2:**
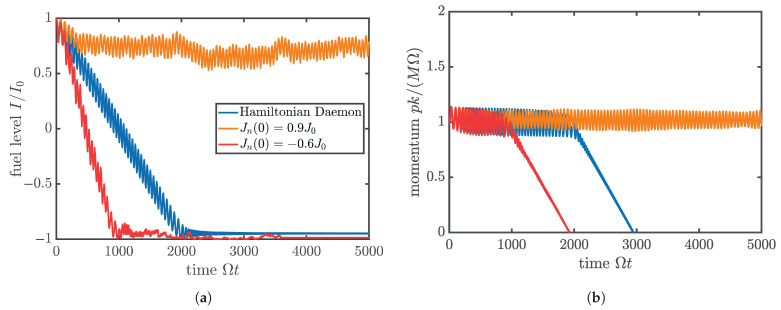
Typical trajectories of (**a**) the active particle’s internal depot “fuel level” $I/I_{0}$ and (**b**) momentum pk/(MΩ), evolved over time according to (5) in an environment without crumbs (blue), and with two different kinds of crumbs. The red and orange cases of crumbs are, respectively: small, low-energy crumbs having $J_{n}\left(0\right)=-0.6J_{0}\gg -I_{0}$ (red) and small, high-energy crumbs having $J_{n}\left(0\right)=+0.9J_{0}\ll I_{0}$ (orange). The red path (with the low-energy crumbs) exits the active phase first, even sooner than in the case with no crumbs, and shows no sign of fuel replenishment. Meanwhile, the small, high-energy crumbs of the orange path sustain the active phase for at least the computed time span. All trajectories have $M=I_{0}k^{2}/\Omega$, γ=0.02Ω, $g=10^{-3}\Omega^{2}/k$ and a=1/k. The crumbs are equidistantly spaced with $q_{n+1}-q_{n}=10/k$.

Some representative trajectories of this system for different *non-thermal* choices of crumb parameters are shown in Figure 2. The most important feature to note is that the Hungry Daemon can indeed successfully harvest energy indefinitely from crumbs which all have high initial energies ($J_{n}\left(0\right)$ close to $+J_{0}$); if it interacts instead with crumbs that all have low initial energies ($J_{n}\left(0\right)$ close to $-J_{0}$), then it actually loses energy to the crumbs, stalls sooner than it would if there were no crumbs, and does not replenish its internal depot.

The particular numerical results shown in Figure 2 do not immediately contradict the intuitive expectation that the active particle will always be able to harvest energy from crumbs if they have enough energy. Indeed, they may seem to support that intuition. However, it is precisely the pattern of high-energy crumbs yielding energy, while low-energy crumbs absorb it, which disproves the intuitive expectation and supports thermodynamics instead.

### 4.2. Interaction with a Single Crumb in a Simple Limit

We can obtain general analytical results for energy harvesting in our Hungry Daemon model by assuming an especially simple limit. This limit will then show us what *can* go wrong with the intuitive argument in favor of perpetual motion from thermal crumbs, such that instead thermodynamics is right. After we understand this issue in the simple limit, we will then argue in the following Section that the problem with the intuitive argument is not restricted to the simple limit, but is instead a general implication of Liouville’s Theorem.

We consider this simple limit by imposing the following special conditions on our Hungry Daemon model parameters:

- The crumbs are much smaller energetically than the particle’s internal depot: $J_{0}\ll I_{0}$, as for the red and orange curves in Figure 2, and as one expects for individual food particles consumed by a small mobile organism;
- The typical interaction duration $\Delta t=a/v_{c}$ between the actively moving particle and a crumb is short compared to 1/γ, so that $H_{C}$ can be neglected over this short interval;
- The effective crumb–depot coupling strength $∼\kappa J_{0}$ is, on the other hand, large enough that the interaction between a crumb and the depot is *not* negligible while $|q-q_{n}|\leq a/2$.

In this simple limit the Hungry Daemon active particle effectively moves just as if there were no crumbs, except that its depot energy ΩI(t) can make a small jump whenever it passes one of the $q_{n}$ crumb locations.

This jump in depot energy occurs over the short interaction time with a crumb, while q(t) is within the interaction range a/2 of a crumb location $q_{n}$. In that time, the crumb reservoir and the particle depot effectively evolve under the simple coupling Hamiltonian

(8) $$H\to H_{\mathrm{int}}=\kappa \sqrt{I_{0}^{2}-I^{2}}\sqrt{J_{0}^{2}-J_{n}^{2}}\cos\left(\delta -\beta_{n}\right).$$

This effective Hamiltonian $H_{\mathrm{int}}$ exactly conserves $I+J_{n}$ so that the jump in I(t) over the short interval Δt is exactly the negative of the change in $J_{n}\left(t\right)$ over this interval. Since $|J_{n}|\leq J_{0}\ll I_{0}$, furthermore, we can neglect the proportionally small possible change in I(t) and treat I(t) as constant throughout this short interval. With this approximation it becomes easy to solve the canonical equations of motion for $J_{n}\left(t\right)$. Defining $t_{n}$ as the moment when the active particle begins interacting with crumb *n*, we obtain

(9) $$\begin{array}{cc} J_{n}\left(t\right) & =J_{n}\left(0\right)\cos\left(\kappa \sqrt{I_{0}^{2}-I^{2}\left(t_{n}\right)}{\left(t-t_{n}\right)}^{+}\right)\\ \; & \;+\sqrt{J_{0}^{2}-J_{n}^{2}\left(0\right)}\sin\left[\beta_{n}\left(t_{n}\right)-\delta \left(t_{n}\right)\right]\sin\left(\kappa \sqrt{I_{0}^{2}-I^{2}\left(t_{n}\right)}{\left(t-t_{n}\right)}^{+}\right), \end{array}$$

where $x^{+}\max\left(x,0\right)$. Since the Daemon active particle always moves in the same direction during its active motion, it will not have interacted with crumb *n* until time $t_{n}$, and since $J_{n}\left(t\right)$ is conserved until the active particle first comes within a/2 of the crumb’s location $q_{n}$, $J_{n}\left(t_{n}\right)$ is simply the initial value of $J_{n}$.

We can safely assume that the relative phase $\beta_{n}\left(t_{n}\right)-\delta \left(t_{n}\right)$ is random, first of all because all $\beta_{n}$ have the same energy until the active particle actually starts interacting with crumb *n*, and secondly because δ(t) evolves rapidly while the active particle moves between crumbs. On average, over an ensemble of many crumbs which all have arbitrary $\beta_{n}-\delta$, therefore, the jump in the active particle’s depot level I(t) after interacting with a crumb with initial energy $J_{n}\left(t_{n}\right)$ for a duration Δt will be

(10) $$\begin{array}{cc} \; & \Delta I_{n}\langle I\left(t_{n}+\Delta t\right)-I\left(t_{n}\right)\rangle =\langle J_{n}\left(t_{n}\right)-J_{n}\left(t_{n}+\Delta t\right)\rangle \\ = & J_{n}\left(0\right)\left[1-\cos\left(\kappa \sqrt{I_{0}^{2}-I^{2}\left(t_{n}\right)}\Delta t\right)\right]≐J_{n}\left(0\right)\left[I_{0}^{2}-I^{2}\left(t_{n}\right)\right]\frac{{\left(\kappa \Delta t\right)}^{2}}{2}, \end{array}$$

where in the last step we have used the assumption that $\kappa I_{0}\Delta t$ is small.

The crucial feature of $\Delta I_{n}$ according to (10) is that it is directly proportional to $J_{n}\left(0\right)$. At least in this simple limit of our particular Hungry Daemon model, therefore, whether the active particle will on average gain energy from a crumb, or lose energy to the crumb, does *not* depend on whether the crumb is initially more energetically excited than the active particle’s depot, but only on whether the crumb is closer in energy to its own energy maximum or to its own energy minimum. We will return to this important point in our discussion below.

### 4.3. Second Law of Thermodynamics

Over a short interaction time Δt, we may assume that the actively moving Daemon keeps moving at close to its active speed Ω/k so that the interaction time can be computed self-consistently as

(11) $$\Delta t=\frac{ka}{\Omega }.$$

Inserting this in (10) and averaging over a thermal distribution of $J_{n}\left(t_{n}\right)$ at temperature *T*, we find the average energy gain $\Delta \bar{E}$ from interacting with crumb *n* at time $t_{n}$ for a duration of Δt during active motion to be

(12) $$\begin{array}{cc} \Delta \bar{E}\left(t_{n},\Delta t\right) & =\int_{-J_{0}}^{J_{0}}\;dJ_{n}\;\frac{e^{-\frac{\Omega J_{n}}{k_{B}T}}}{2\frac{k_{B}T}{\Omega }\sinh\frac{\Omega J_{0}}{k_{B}T}}\;\Omega \Delta I_{n}\\ \; & =\frac{{\left(\kappa \Delta t\right)}^{2}\Omega J_{0}}{2}\left(\Theta -\coth\Theta^{-1}\right)\left(I_{0}^{2}-I^{2}\left(t_{n}\right)\right), \end{array}$$

with the dimensionless temperature $\Theta =\frac{k_{B}T}{\Omega J_{0}}$. The dependence of $\Delta \bar{E}$ on both Δt and $t_{n}$ should emphasize that it represents the energy transfer with crumb *n* at time $t_{n}$ after an interaction duration of Δt, while the bar reminds us that it was averaged over the distribution of the crumb fuel levels.

We can immediately note that $\Delta \bar{E}\left(t_{n},\Delta t\right)$ is *negative* for all finite positive *T*, because cothx>1/x for all x>0. On average the Hungry Daemon does *not* succeed in harvesting energy from thermally excited crumbs at any positive temperature. Instead, its internal depot actually loses energy to the crumbs, on average. At least within the simple limit and its approximations, therefore, mechanics agrees with thermodynamics that perpetual motion cannot be powered solely by a thermal source with a single positive temperature.

We can confirm that our approximations which yielded this result are indeed accurate by further considering that the Daemon moves actively through an ensemble of crumbs spaced Δq apart, and therefore interacts with crumbs at an average rate Δqk/Ω. As a result, the depot loses energy at an average rate of $\Delta \bar{E}\left(t,\Delta t\right)\Omega /\left(\Delta qk\right)$ to the crumbs. Including the fuel level decrease −Mg/k needed to sustain activate motion (see Section 3), the average total rate at which the Daemon’s internal depot must be changing should then be

(13) $$\dot{I}\left(t\right)=-\frac{Mg}{k}+\frac{\Omega }{\Delta qk}\Delta \bar{E}\left(t,\Delta t\right),$$

which is a Ricatti equation for I(t). If we start the Daemon with a full internal depot, $I\left(0\right)=I_{0}$, then it runs actively until the time $t^{*}$ at which $I\left(t^{*}\right)=-I_{0}$, and we can find an analytical expression for $t^{*}$ by integrating (13). Multiplying by the power MgΩ/k applied by the internal depot against the external force yields the average total work $\Delta {\bar{E}}_{\mathrm{tot}}$ done by the Daemon in its active motion:

(14) $$\Delta {\bar{E}}_{\mathrm{tot}}=\frac{Mg\Omega }{k}t^{*}=\frac{Mg\Omega }{k}I^{-1}\left(-I_{0}\right).$$

**Figure 3:**
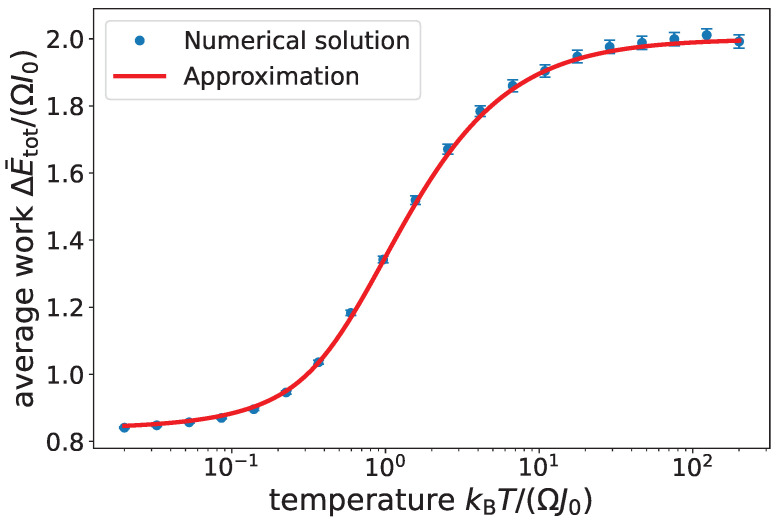
Average total work $\Delta {\bar{E}}_{\mathrm{tot}}$ of the Daemon approximated according to (13) (red curve) together with the results of a Monte Carlo computation with 1000 trials (blue points with 95 % confidence intervals as error bars) in dependence on temperature of the crumb energy distribution. The close agreement is unsurprising for the specific case shown, which has $M\Omega /(k^{2}I_{0})=1$, γ=0.02Ω, $g=10^{-3}\Omega^{2}/k$, $J_{0}=0.05I_{0}$, a=1/k, Δq=10/k, $\kappa =\Omega /I_{0}$$I\left(0\right)=I_{0}$, and p(0)=MΩ/k.

This analytical result for the Daemon’s work output, based on our approximate analysis in this section, is compared in Figure 3 with a numerically exact solution to the full equations of motion for our system for ensembles of random crumbs obtained by sampling thermal distributions at different temperatures. The agreement is close.

## 5. Discussion

### 5.1. Summary: The Hungry Daemon Is Not a Perpetual Motion Machine of the Second Kind

The intuitive expectation for the Hungry Daemon was that it would surely gain energy from crumbs, at least on average, if they had more energy or higher temperature than it did, thus enabling it to continuously extract energy from its environment as soon as its fuel level is low enough. This expectation was based on the standard thermodynamical assumption that a system which interacts with a heat bath will tend to equilibrate with the bath, so that its own temperature approaches that of the bath. This seemingly reasonable expectation would imply that if the energy of the Daemon’s depot fell below the crumb temperature then it would on average steadily gain energy from the crumbs—and in this case the Daemon could keep moving against the external force forever, as long as the crumbs were hot enough, and frequent enough, to supply energy fast enough.

This expectation was, however, in conflict with Thermodynamics, since such continuous energy extraction from a thermal reservoir to do continuous work would explicitly represent a Perpetual Motion Machine of the Second Kind, being in direct conflict with the Kelvin–Planck formulation of the Second Law that we have cited above. The expectation that the Daemon’s depot would tend to equilibrate with the crumbs in the usual way may correspond with typical thermodynamical situations, but since in this case this typical expectation actually breaks the Second Law, something that does not fit with typical Thermodynamics must be going on here in any case: either the Daemon depot does not equilibrate with the crumbs as expected, or else the Second Law does not hold. So the expectation about equilibration on the part of the depot must be re-examined.

In fact it is the expectation of equilibration by the depot which fails in our case, and not the Second Law. The Hungry Daemon’s depot interacts with the crumbs, but its specific kind of interaction with them, individually in succession, with steady motion against an external force in-between, turns out not to be a form of interaction which would make the Daemon’s depot equilibrate with the crumbs while keeping the Daemon’s center of mass co-ordinate in a non-equilibrium state of steady uphill motion at a near-constant speed. We have now shown, strictly based on the Laws of Mechanics, that our Hungry Daemon can, in fact, never do what the Second Law forbids. It thus obeys the Second Law, even though it has been described microscopically and without dissipation.

The appeal of our result is that its origin can be traced all the way back to the microscopic interaction of the particle with a single crumb. According to (10), the direction of the average energy exchange between the two depends only on the sign of the crumb’s fuel level variable $J_{n}$, and *not* on the difference between the daemon’s and crumb’s fuel level, as one would expect from a phenomenological relaxation approach. In consequence, when the crumb ensemble is in thermal equilibrium at positive temperature, the average fuel level $J_{n}$ of a crumb is negative, implying that the daemon more often loses energy to the crumbs than gaining energy from them. Its fuel trajectory therefore resembles more the red than the orange curve in Figure 2. Under these circumstances, the Hungry Daemon cannot replenish its reservoir in a periodic process and must eventually stop moving.

### 5.2. Generality?

Our analytical and numerical results have only explicitly described one simple limit of one artificial model system. By showing what can go wrong with intuitive expectation, however, these model calculations have indicated the general mechanical reason why the Second Law must be valid for a large class of possible energy-harvesting active particles. The mechanical reason for the Second Law, at least in this class of systems, is Liouville’s theorem.

For crumbs having any possible Hamiltonian $H_{n}$, any positive-temperature phase space distribution

(15) $$P_{n}\left({\vec{Q}}_{n},{\vec{P}}_{n}\right)=Z_{n}^{-1}\exp\left(-\frac{H_{n}\left({\vec{Q}}_{n},{\vec{P}}_{n}\right)}{k_{B}T}\right)$$

can be considered as an ensemble of sub-ensembles that each have uniform probability for all energies below some maximum energy E:

(16) $$P\left({\vec{Q}}_{n},{\vec{P}}_{n}\right)=\int \;dE{\tilde{P}}_{n}\left(E\right)\theta \left(H_{n}-E\right)$$

for some sub-ensemble probability distribution ${\tilde{P}}_{n}\left(E\right)$.

For each such sub-ensemble of all crumbs, each uniformly filling the phase space of each crumb *n* from the ground state to some $E_{n}$, and for any initial state of the active particle and its internal depot, the entire system of crumbs and active particle fills a certain total phase space volume uniformly. This will be true even for greatly generalized versions of our simple model, with arbitrary forms of interaction between active particle and crumbs.

Liouville’s theorem then says that this initial phase space volume (actually the product of all the individual subsystem volumes) can never change under time evolution. Yet, if the active particle were to consistently extract energy from the crumbs, on average, their ensembles would have to be compressed downwards from $E_{n}$ to their ground states. If the energy harvesting could continue indefinitely, then this shrinking of the initial phase space volume would have to continue indefinitely as more and more crumbs and their energies drained—and therefore their phase space volumes.

The internal depot of an active particle is finite, and can only hold a finite amount of energy—or phase space volume. The momentum of the active particle likewise remains bounded, and its position is supposed to be systematically moving in time if perpetual energy harvesting is happening, not diffusing over an exponentially increasing range. So there is no way for the phase space volume of the active particle itself to increase sufficiently to make the steady decrease of crumb phase space volume consistent with Liouville’s theorem.

Phase space volume and Liouville’s theorem thus function for active particle energy harvesting as the microscopic, mechanical proxies for entropy and the Second Law. This is the relationship which is always suspected in seeking the microscopic underpinnings of the Second Law, but it cannot always be confirmed so explicitly. The impossibility of perpetual energy harvesting from thermally excited crumbs, which we found in our particular model, is indeed clearly general. As a new variant of a Maxwell’s Demon, the Hungry Daemon has indeed shed some light on the relationship between thermodynamics and microscopic mechanics.

### 5.3. Harvesting Can Succeed if There Is Energetic Inversion

The Hungry Daemon also confirms microscopically and mechanically something that is generally assumed about energy harvesting: that harvesting does *not* actually violate thermodynamics, because an environment which offers usable food or fuel is one that has some kind of energetic population inversion, with at least some higher energy states being more common than they could be in a positive-temperature thermal state. For crumbs with energy bounded from above as well as from below, this kind of energetic inversion is possible even within the canonical ensemble by taking a *negative* temperature. For negative temperatures, $\Delta I_{n}$ in Equation (10) can be positive, and for a given opposing force −Mg and rate of encountering crumbs, there can be a sufficiently large negative *T* for the crumbs at which the Hungry Daemon does replenish its depot fast enough to keep moving indefinitely. And with negative temperature, the Second Law does *not* forbid perpetual motion, since extracting energy then *increases* entropy.

Crumbs with Boltzmann-distributed energies at a negative *T* are not necessarily realistic, even if the crumb energy is bounded from above, but the formal possibility of perpetual motion through energy harvesting from negative-temperature crumbs indicates that energetic population inversion of any kind can be sufficient to permit energy harvesting. This kind of energetic inversion is indeed realistic when one considers that energy sources such as ATP molecules are present in intracellular environments at concentrations far above equilibrium. With negative temperature or with any more general kind of inversion, the sub-ensemble argument that we have just presented must be revised to consider sub-ensembles of uniform probability *above* a threshold energy E, instead of below. The argument from Liouville’s theorem then changes direction to forbid steady energy loss to the crumbs, but allow steady harvesting. Liouville’s theorem does not allow uniformly filled phase space volumes to increase any more than it allows them to decrease, but it allows an initially uniform ensemble to become dilute, being made porous with swirls, so that it fills a larger volume in a coarse-grained sense. It does not allow a uniformly filled phase space volume to compress. So energy harvesting *is* allowed to continue indefinitely if the crumbs have sufficient energetic inversion.

In this sense the Hungry Daemon model has also shed light on energy harvesting by highlighting the special thermodynamical character of food. Food must not merely be energy, but thermodynamically available energy: it must have some form of energetic population inversion, with higher food energies appearing more often than they would in equilibrium.

### 5.4. Energy-Harvesters Versus Heat Engines

In order to satisfy Liouville’s theorem, energy extraction must conserve action. Re-examining our impossibility proof from Liouville’s theorem, above, we can see that energy harvesting from a positive-temperature source could become possible after all if the active particle would just divert a bit of its harvested energy to steadily excite some additional, ancillary degrees of freedom in its environment which were previously less energetically excited than the crumbs. The amount of diverted energy that would be needed to satisfy Liouville’s theorem, and enable steady energy harvesting, does not need to be as much as the energy which is taken from the crumbs. It just has to be enough to expand the phase space volume of those ancillary degrees of freedom enough to satisfy Liouville.

In other words, we can harvest energy from a single hot bath if we can expel heat to a colder bath. We have invented the heat engine. Or rather, of course, we have recognized that having access to two baths at different temperatures, enabling a heat engine, is a particular form of the kind of uneven distribution of energies in the environment that can also be exploited for energy harvesting from food or fuel.

And, now that we have seen how Liouville’s theorem supports Thermodynamics when it applies, we can further see how dissipation can be an advantage rather than an obstacle for naturally occurring active particles. Adding dissipation to our system would implicitly be adding a further reservoir, which could be colder than the reservoir of positive-temperature crumbs, and thus allow continuing motion against friction without any conflict with Thermodynamics. A world without any friction might not be one in which we were free from thermodynamic constraints, but rather one in which it was harder to deal with the constraints of Liouville’s theorem.

## Data Availability

The original contributions presented in this study are included in the article. Further inquiries can be directed to the corresponding author.

## Abbreviations

The following abbreviations are used in this manuscript:

*a*	crumb width
$\beta_{n}$	crumb phase variable
γ	coupling constant between daemon depot and motion
δ	depot phase variable
$\Delta \bar{E}$	average energy transfer from a crumb to the daemon depot
$\Delta {\bar{E}}_{\mathrm{tot}}$	average energy transfer from all crumbs encountered during the active phase to the daemon depot
E	system energy
*g*	acceleration
$H_{C}$	coupling Hamiltonian
$H_{D}$	daemon Hamiltonian
$H_{I}$	depot Hamiltonian
$H_{M}$	motional Hamiltonian
$H_{n}$	crumb Hamiltonian
*I*	daemon fuel level coordinate
$I_{*}$	daemon fuel level at the end of the active phase
$I_{0}$	daemon maximal fuel level
$\bar{I}$	daemon fuel level averaged over the tank phases δ(0) and $\beta_{n}\left(0\right)$ as well as over the thermally distributed crumb fuel levels $J_{n}\left(0\right)$
$J_{n}$	crumb fuel level coordinate
$J_{n}\left(0\right)$	crumb initial fuel level
$J_{0}$	crumb maximal fuel level
*k*	angular wave number
$k_{B}$	Boltzmann constant
κ	coupling constant between depot and crumb
*M*	daemon mass
*p*	daemon momentum
$p_{c}$	critical momentum
*q*	daemon position
*P*	probability for a crumb to have an energy below some threshold E
$P_{n}$	probability distribution in phase space for crumb *n*
${\tilde{P}}_{n}\left(E\right)$	sub-ensemble probability density in phase space for crumb *n*
${\vec{P}}_{n}$	momentum coordinate of crumb *n*
$q_{n}$	crumb position
Δq	crumb spacing
${\vec{Q}}_{n}$	position coordinate of crumb *n*
*t*	time
$t_{n}$	moment of begin of interaction between daemon and crumb
Δt	interaction duration
$t_{m}$	duration of active phase
*T*	temperature
$v_{c}$	critical velocity
Ω	frequency
$Z_{n}$	canonical partition function for crumb *n*

## Appendix A. Equations of Motion

The resulting EOM from the Hamiltonian (5) are given by

(A1) $$\begin{array}{cc} \dot{q} & =\frac{\partial H}{\partial p}=\frac{p}{M}, \end{array}$$

(A2) $$\begin{array}{cc} \dot{p} & =-\frac{\partial H}{\partial q}=-Mg-k\gamma \sqrt{I_{0}^{2}-I^{2}}\sin\left(kq-\delta \right), \end{array}$$

(A3) $$\begin{array}{cc} \dot{\delta } & =\frac{\partial H}{\partial I}=\Omega +\gamma \frac{I}{\sqrt{I_{0}^{2}-I^{2}}}\cos\left(kq-\delta \right)-\kappa \sum_{n=1}^{N}\theta (a/2-|q-q_{n}\left|\right)I\sqrt{\frac{\left(J_{0}^{2}-J_{n}^{2}\right)}{\left(I_{0}^{2}-I^{2}\right)}}\cos\left(\delta -\beta_{n}\right), \end{array}$$

(A4) $$\begin{array}{cc} \dot{I} & =-\frac{\partial H}{\partial \delta }=\gamma \sqrt{I_{0}^{2}-I^{2}}\sin\left(kq-\delta \right)+\kappa \sum_{n=1}^{N}\theta (a/2-|q-q_{n}\left|\right)\sqrt{\left(J_{0}^{2}-J_{n}^{2}\right)\left(I_{0}^{2}-I^{2}\right)}\sin\left(\delta -\beta_{n}\right), \end{array}$$

(A5) $$\begin{array}{cc} {\dot{\beta }}_{n} & =\frac{\partial H}{\partial J_{n}}=\Omega -\kappa \theta (a/2-|q-q_{n}\left|\right)J_{n}\sqrt{\frac{I_{0}^{2}-I^{2}}{J_{0}^{2}-J_{n}^{2}}}\cos\left(\delta -\beta_{n}\right), \end{array}$$

(A6) $$\begin{array}{cc} {\dot{J}}_{n} & =-\frac{\partial H}{\partial \beta_{n}}=-\kappa \theta (a/2-|q-q_{n}\left|\right)\sqrt{\left(J_{0}^{2}-J_{n}^{2}\right)\left(I_{0}^{2}-I^{2}\right)}\sin\left(\delta -\beta_{n}\right). \end{array}$$
